# *APOE* ε4 Allele Distribution and Association With Scores of Subjective Cognitive Decline Questionnaire 9 in a Large Chinese Memory Clinic Cohort

**DOI:** 10.3389/fnins.2022.829031

**Published:** 2022-06-03

**Authors:** Lixiao Hao, Jianguo Jia, Yue Xing, Ying Han

**Affiliations:** ^1^Department of General Practice, Xuanwu Hospital of Capital Medical University, Beijing, China; ^2^Radiological Sciences, Division of Clinical Neuroscience, Queen’s Medical Centre, University of Nottingham, Nottingham, United Kingdom; ^3^Department of Neurology, Xuanwu Hospital of Capital Medical University, Beijing, China; ^4^National Clinical Research Center for Geriatric Disorders, Beijing, China; ^5^Center of Alzheimer’s Disease, Beijing Institute for Brain Disorders, Beijing, China

**Keywords:** *APOE* ε4, distribution, demography, SCD-Q9, discrimination

## Abstract

**Background:**

Previous reports on *APOE* ε4 allele distribution in different populations have been inconclusive. The Subjective Cognitive Decline-Questionnaire 9 (SCD-Q9) was developed to identify those at risk of objective cognitive impairment [OCI; including mild cognitive impairment (MCI) and dementia groups), but its association with *APOE* ε4 and discriminatory powers for SCD_*with subtle cognitive decline*_ (SCDs) and OCI in memory clinics are unclear.

**Objectives:**

To investigate demographic distribution of *APOE* ε4, its association with SCD-Q9 scores, and its ability to discriminate SCDs and OCI groups from normal control (NC).

**Methods:**

A total of 632 participants were recruited (NC = 243, SCDs = 298, OCI = 91). *APOE* ε4 allele distribution and association with SCD-Q9 scores were calculated and the effects on cognitive impairment were analyzed. Receiver operating characteristic (ROC) analysis was applied to identify discriminatory powers for NC, SCDs, and OCI.

**Results:**

Total *APOE* ε4 frequency was 13.1%. This did not vary by demography but was higher in patients with OCI. The SCD-Q9 scores were higher in *APOE* ε4 carriers than non-carriers in the OCI group. The area under the curve (AUC) for discriminating from OCI using *APOE* ε4 were 0.587 and 0.575, using SCD-Q9 scores were 0.738 and 0.571 for NC and SCDs groups, respectively. When we combined *APOE* ε4 and SCD-Q9 scores into the model, the AUC increased to 0.747 for discriminating OCI from NC. However, when OCI group was split into MCI and dementia groups, only total SCD-Q9 score was the independent affecting factor of MCI.

**Conclusion:**

This study demonstrated that the distribution of *APOE* ε4 alleles did not vary with different demographic characteristics in a large-scale cohort from a memory clinic. *APOE* ε4 alleles may be associated with scores of SCD-Q9 reflecting the degree of cognitive complaints but their additional contribution to SCD-Q9 scores is marginal in discriminating between NC, SCDs, and OCI.

## Introduction

Previous studies have reported that mutation of Apolipoprotein E (*APOE*) and regulation of its expression have an important connection with Alzheimer’s disease (AD) dementia (Kim and Tsai, 2009) because of the pivotal role of *APOE* in lipoprotein metabolism in the brain. Among the three alleles of *APOE* (ε2, ε3, and ε4), presence of ε4 can increase the risk of AD by approximately 3- (single allele) to 15-fold (double alleles) (Saunders et al., 1993; Kim and Tsai, 2009; Koffie et al., 2012).

An earlier meta-analysis and review of the distributions of *APOE* alleles showed that *APOE* ε4 alleles were the second most common allele (besides *APOE* ε3) (Farrer et al., 1997; Abondio et al., 2019), but distributions vary with age, gender, and ethnicity (Eisenberg et al., 2010; Kern et al., 2015; Le Couteur et al., 2020). Among cognitively normal subjects aged 21–97 years, *APOE* ε4 carriers were younger than non-carriers, but no significant differences were found in education level and gender (Caselli et al., 2009). Farrer et al. (1997) demonstrated that *APOE* ε4 was associated with the risk of AD from 40 to 90 years, but the relationship diminished after 70 years of age. They also reported that the frequency of the *APOE* ε4 allele in males was lower than that in females among the elderly without dementia (Lehmann et al., 2006). In addition, previous studies investigating different ethnic groups revealed that Asian populations might have lower *APOE* ε4 frequencies than Oceania in the natural population (Singh et al., 2006; Eisenberg et al., 2010), and Chinese cohorts presented relatively low frequencies of *APOE* ε4 alleles (Katzman et al., 1997), but the conclusions need to be confirmed by more research.

More recently, researchers have also focused on understanding the relevance of *APOE* ε4 prevalence and the early stages of cognitive impairment, such as subjective cognitive decline (SCD), which is an intermediate stage between mild cognitive impairment (MCI) and normal cognition. A systematic review (Ali et al., 2018) (*n* = 36 articles) showed that the frequency of *APOE* ε4 was significantly lower in healthy controls than in groups with objective cognitive impairment (OCI), including MCI and AD dementia, but showed no difference in the SCD group, suggesting that *APOE* ε4 may not be directly related to the development of SCD. However, another earlier meta-analysis included a total of 28 studies that indicated a weak positive correlation between *APOE* ε4 and SCD (Zhang et al., 2017). This inconsistency may be due to different study designs (e.g., age) and the smaller sample size, especially in memory clinics, most of which evaluated less than 100 subjects (Striepens et al., 2010; Fortea et al., 2011). However, memory clinics, as the primary setting for individuals with memory complaints seeking care, are crucial for the early identification of those at risk of cognitive decline.

Additionally, since the standardization of SCD with cognitive complaints but unimpaired cognition (Jessen et al., 2014) in 2014, there is growing evidence demonstrating that subtle cognitive decline is already present in populations with SCD (Kielb et al., 2017; Jessen et al., 2018; Hao et al., 2019). To improve the likelihood of preclinical AD diagnosis, a more sensitive and reliable neuropsychological standard for subtle cognitive decline (Jak/Bondi) was developed in 2015 (Edmonds et al., 2015). However, the relationship between SCD_*with subtle cognitive decline*_ (SCDs) diagnosis based on the two updated criteria and *APOE* ε4 within a large cohort has not been reported.

Finally, in recent years the SCD-Questionnaire 9 (SCD-Q9), developed as an easy and quick screening tool, was used to identify patients with SCD at risk of OCI at an early stage using advanced statistical methods (Gifford et al., 2015). Since its development, several studies (Alber et al., 2018; Bott et al., 2018; Kumar et al., 2018; Hao et al., 2020) have applied it to assess changes in memory complaints and defined SCD. Previous evidence has also demonstrated the links between the subjective observations included in SCD-Q9 and objective pathological alterations (Amariglio et al., 2012). *APOE* ε4 as an objective biomarker of AD has been universally accepted. However, the association between SCD-Q9 scores and *APOE* ε4 and their predictive powers for OCI with SCD complaints are not clear. Elucidation of this association would help us better understand the biomarkers of AD reflected by subjective cognitive complaints. The combination of the two methods may help us to more quickly and accurately identify early AD patients and could also reduce the economic burden of the society and families.

Therefore, our current investigation studies larger cohorts in memory clinic settings, mainly aimed to (1) investigate the distribution characteristics of *APOE* ε4 alleles in different demography and cognitive impairment groups diagnosed based on updated criteria (SCD-I) (Jessen et al., 2014) and Jak-Bondi (Edmonds et al., 2015); (2) analyze the association of *APOE* ε4 and scores of SCD-Q9 reflecting cognitive impairment; and (3) assess the discriminatory powers of *APOE* ε4 alleles themselves and their combination with scores of SCD-Q9 for diagnosing cognitive impairment.

## Materials and Methods

### Participants

#### Subject Recruitment

Six hundred thirty-two individuals participated our study, SCDs, MCI, and AD dementia patients were recruited at first routine visits to the memory clinic of the Neurology Department, Xuanwu Hospital, Capital Medical University and normal control (NC) subjects were recruited from communities in Beijing, China from March, 2017 to January, 2020. The details of the study, including its purpose, procedure and contact information, was advertised in the memory clinic and via broadcasting at large-scale gatherings in the communities. People were asked for their consent to join the study.

#### Study Procedure and Subject Selection Criteria

All the subjects underwent a series of clinical and standardized neuropsychological evaluations, including the sociodemographic characteristics, medical history, lifestyles, and a neuropsychological test battery which contains Chinese version of Mini-Mental State Examination (MMSE) (Katzman et al., 1988), Montreal Cognitive Assessment – Basic (MoCA-B) (Chen et al., 2016b), Clinical Dementia Rating Scale (CDR) (Morris, 1993), Activities of Daily Living (ADL) (He et al., 1990), Memory: Auditory Verbal Learning Test (AVLT)-Long Delay Recall and Recognition (Guo et al., 2007), Executive function: Shape Trail Making Test-A and B (STT-A and STT-B) (Zhao et al., 2013b), Language: Animal Fluency Test (AFT) (Zhao et al., 2013a), Boston Naming Test (BNT) (Guo et al., 2006), Hamilton Anxiety Scale (HAMA) (Tang and Zhang, 1984), and Hamilton Depression Scale (HAMD) (Hamilton, 1980).

##### Inclusion Criteria for All Subjects

Jak/Bondi criteria as follows (Bondi et al., 2014; Edmonds et al., 2015) were used for the diagnosis of MCI and SCDs.

Mild cognitive impairment was assigned when (1) the answer needed to be “yes” to the question “Do you have a problem with your memory?”; (2) scores of two measures in the same cognitive domain were >1.0 standard deviation (SD) below the normative mean; or (3) scores of at least one measure in each of the three cognitive domains (Memory, Execution, and Language) were >1.0 SD below the normative mean; (4) failure to meet the criteria of dementia; and (5) ADL had to be normal.

For the diagnosis of SCDs, the following requirement was to be met: (1) the answers needed to be “yes” to both of the questions “Do you have a problem with your memory?” and “Are you concerned about your memory?”; (2) subtle cognitive decline was observed in the neuropsychological examination, indicated by the decreased score of two measures in different cognitive domains (>1.0 SD below the normative mean); (3) failure to meet the criteria of MCI; and (4) ADL was normal.

The diagnosis of mild AD dementia fulfilled standardized diagnostic criteria (Mckhann et al., 1984; American Psychiatric Association, 1994; Dubois et al., 2007): (1) met the diagnostic criteria of dementia; (2) gradual and progressive decline in memory function over more than 6 months; (3) impaired episodic memory revealed by the objective testing listed above; (4) impaired basic and elementary functioning for ADL; (5) CDR = 1; and (6) hippocampal atrophy confirmed by structural magnetic resonance imaging (MRI).

##### Groupings

The population was divided into three groups according to these listed diagnostic criteria: (1) NC was assigned when participants did not have SCD complaints (the answers needed to be “no” to both of the questions “Do you have a problem with your memory?” and “Are you concerned about your memory?”) and mild AD dementia, MCI, or SCDs, and had normal ADL scores; (2) SCDs group; and (3) the OCI group included people who were diagnosed with MCI and mild AD dementia.

##### Exclusion Criteria for All Subjects

(a) A history of stroke; (b) severe depression (HAMD >30), and other psychiatric disorders or current psychotropic drugs treatment; (c) other central nervous system diseases that could cause cognitive decline (e.g., brain tumors, Parkinson’s disease, encephalitis, or epilepsy); (d) other systemic diseases which could cause cognitive decline (e.g., alcoholism, thyroid dysfunction, severe anemia, syphilis, HIV, or vitamin B_12_ abnormalities); (e) a history of psychosis or congenital mental growth retardation; (f) cognitive decline caused by traumatic brain injury; (g) use of anti-dementia agents in SCDs, MCI, and; or (h) those who could not complete neuropsychological tests or with contraindication to MRI.

#### *APOE* Genotyping

DNA sequences for each subject were extracted for SNPs rs7412 and rs429358 from the *APOE* ε2/ε3/ε4 haplotype. *APOE* was genotyped using the standard Sanger sequencing method (Sangon, Shanghai, China) with the following primers: 5′-ACGCGGGCACGGCTGTCCAAGG-3′ (forward) and 5′-GGCGCTCGCGGATGGCGCTGA-3′ (reverse). *APOE* was amplified using the following conditions: 1 cycle of 98°C for 10 s, 35 cycles of 72°C for 5 s, and 1 cycle of 72°C for 5 min. PCR was performed in a final volume of 30 μl containing 10 pmol of forward and reverse primers, and 50 ng of genomic DNA template using PrimeSTAR HS DNA Polymerase with the GC Buffer (Takara Bio).

### Statistical Analysis

We conducted all analyses using the Statistical Package for the Social Sciences version 17.0 (SPSS Inc., Chicago, IL, United States). Descriptive statistics (*APOE* alleles and scores of SCD-Q9) were calculated by percentages or mean ± SD (*x* ± *S*) or median (percentile 25, 75). The x^2^ or *T*-test or Mann–Whitney test was used to assess group differences, and *p* < 0.05 was considered to be statistically significant. For three groups comparison, *p* < 0.05 was considered to be statistically significant and corrected *p*’ value (*p* < 0.017) was used in the partitions of Pearson’s Chi-square statistics. To examine the potential affecting factors of SCDs and OCI, we performed univariate and binary logistic regression analysis (Supplementary Materials 1). More specifically, we used *APOE* ε4 alleles and scores of SCD-Q9 that significantly differed between two groups as the independent variables, and “diagnosis” as the dependent variable. Besides, odds ratios (ORs) were calculated for the two variables. *p* < 0.05 was required for variables to be in the model. Finally, we obtained the receiver operating characteristic (ROC) curves and calculated area under the curves (AUCs) for the factors.

## Results

### Distribution of *APOE* ε4 Alleles and Genotypes in the Total Population

In total, 632 individuals were recruited in our study, including 218 (34.5%) males and 414 (65.5%) females, and the mean age and education years were 65.4 ± 6.76 and 12.4 ± 3.21 years, respectively. The proportions of *APOE* genotypes were listed in Table 1.

**TABLE 1:** The distribution of *APOE* ε4 alleles and genotypes in total population.

Subtypes	*N*	Percentage (%)
*APOE* ε2/2	5	0.8
*APOE* ε2/3	78	12.3
*APOE* ε3/3	393	62.2
*APOE* ε2/4	18	2.8
*APOE* ε3/4	128	20.3
*APOE* ε4/4	10	1.6
*APOE* ε2	106	8.4
*APOE* ε3	992	78.5
*APOE* ε4	166	13.1

The proportions of *APOE* ε3/4 and ε2/4 were 20.3% and 2.8%, respectively. The proportion of homozygous ε4 was <2.0%. For the frequencies of the *APOE* alleles ε4 was 13.1% (see Table 1).

### The Demography of *APOE* ε4 Carriers and Non-carriers

For age, gender, and years of education, we did not find significant differences between *APOE* ε4 carriers and non-carriers (*p* > 0.05) (see Table 2).

**TABLE 2:** The distribution of *APOE* ε4 carriers and non-carriers in different demography.

Demography	Non-carriers*n* (%)	Carriers *n* (%)	*p*
Gender			0.130
Male	172 (78.9)	46 (21.1)	
Female	304 (73.4)	110 (26.6)	
Age*			0.969
≤65 years	248 (75.4)	81 (24.6)	
>65 years	228 (75.2)	75 (24.8)	
Education*			0.720
≤12 years	276 (74.8)	93 (25.2)	
>12 years	200 (76.0)	63 (24.0)	

*For age and education, we selected the mean as the cut-off value to stratify the group.

### Distribution of *APOE* ε4 and Subjective Cognitive Decline-Questionnaire 9 Scores of Carriers and Non-carriers in Normal Control, SCDs, and Objective Cognitive Impairment Groups

The results showed that the difference in the proportion of *APOE* ε4 carriers among the three groups (NC, SCDs, and OCI) was significant (*p* = 0.004). Further pairwise comparison showed that the OCI group had a higher proportion of *APOE* ε4 carriers than the NC (*p* = 0.001) and SCDs groups (*p* = 0.005) at corrected test level *p*′, but no significant difference was found between the other two groups (*p* = 0.487) (see Table 3). When the total population was divided into male and female subgroups, the latter showed consistent results with the total population (see Table 4), but we did not find any significant differences among NC, SCDs, and OCI groups in the male subgroup (see Table 5).

**TABLE 3:** Distribution of *APOE* ε4 and SCD-Q9 scores of carriers and non-carriers in NC, SCDs, and OCI groups.

Groups	Non-carriers *n* (%)	Carriers *n* (%)	*p*	SCD-Q9 (*x* ± *s*)		*p*
				Non-carriers	Carriers	
Total population	476 (75.3)	156 (24.7)	–	4.24 ± 2.17	4.63 ± 2.20	0.057
NC (*n* = 243)	192 (79.0)	51 (21.0)	0.004	3.26 ± 2.28	3.16 ± 2.21	0.766
SCDs (*n* = 298)	228 (76.5)	70 (23.5)		4.92 ± 1.66	5.00 ± 1.57	0.703
OCI (*n* = 91, MCI = 77, AD dementia = 14)	56 (61.6)	35 (38.4)		4.87 ± 2.38	6.01 ± 2.10	0.022

*NC, normal control; SCDs, subjective cognitive decline_with subtle cognitive decline_; OCI, objective cognitive impairment; SCD-Q9, Subjective Cognitive Decline-Questionnaire 9; MCI, mild cognitive impairment; AD, Alzheimer’s disease.*

**TABLE 4:** Distribution of *APOE* ε4 and SCD-Q9 scores of carriers and non-carriers in NC, SCDs, and OCI groups in females.

Groups	Non-carriers *n* (%)	Carriers *n* (%)	*p*	SCD-Q9 (*x* ± *s*) percentile 50 (percentile 25, 75)		*p*
				Non-carriers	Carriers	
Total females	304 (73.4)	110 (26.6)	–	4.5 (3.0, 6.0)	4.8 (3.0, 6.0)	0.585
NC (*n* = 144)	109 (75.7)	35 (24.3)	<0.001	3.5 (2.0, 5.3)	3.0 (1.0. 4.5)	0.159
SCDs (*n* = 225)	173 (76.9)	52 (23.1)		5.0 (4.0, 6.0)	5.0 (4.0, 6.0)	0.937
OCI (*n* = 45, MCI = 38, AD dementia = 7)	22 (48.9)	23 (51.1)		5.3 (2.9, 6.6)	6.0 (5.5, 7.5)	0.042

*NC, normal control; SCDs, subjective cognitive decline_with subtle cognitive decline_; OCI, objective cognitive impairment; SCD-Q9, Subjective Cognitive Decline-Questionnaire 9; MCI, mild cognitive impairment; AD, Alzheimer’s disease.*

**TABLE 5:** Distribution of *APOE* ε4 and SCD-Q9 scores of carriers and non-carriers in NC, SCDs, and OCI groups in males.

Groups	Non-carriers *n* (%)	Carriers *n* (%)	*p*	SCD-Q9 (*x* ± *s*) percentile 50 (percentile 25, 75)		*p*
				Non-carriers	Carriers	
Total males	172 (78.9)	46 (21.1)	–	4.0 (2.0,5.0)	4.8 (3.4, 6.1)	0.032
NC (*n* = 99)	83 (83.8)	16 (16.2)	0.260	2.5 (1.0, 4.5)	3.5 (1.0, 5.4)	0.220
SCDs (*n* = 73)	55 (75.3)	18 (24.7)		5.0 (3.5, 6.0)	4.4 (5.0. 6.0)	0.709
OCI (*n* = 46, MCI = 39, AD dementia = 7)	34 (73.9)	12 (26.1)		5.0 (3.4, 7.1)	6.5 (3.3, 8.0)	0.275

*NC, normal control; SCDs, subjective cognitive decline_with subtle cognitive decline_; OCI, objective cognitive impairment; SCD-Q9, Subjective Cognitive Decline-Questionnaire 9; MCI, mild cognitive impairment; AD, Alzheimer’s disease.*

For SCD-Q9 scores, we also found that *APOE* ε4 carriers scored higher than non-carriers in the OCI group (*p* = 0.022). However, there were no significant differences in SCD-Q9 scores in the total population (*p* = 0.057), NC (*p* = 0.766), and SCDs groups (*p* = 0.703) between ε4 carriers and non-carriers (see Table 3).Then, we divided the total population into female and male subgroups. For the female subgroup, the results were consistent with the total population (see Table 4). However, a significant difference was found in SCD-Q9 scores between ε4 carrier and non-carriers in the males (*p* = 0.032), which was different from the female subgroup and the total population. Also, we did not find any significant difference in SCD-Q9 scores between ε4 carriers and non-carriers in different cognitive groups, including NC (*p* = 0.220), SCDs (*p* = 0.709), and OCI groups (*p* = 0.275) (see Table 5).

### Logistic Regression Models for Normal Control, SCDs, and Objective Cognitive Impairment Groups

The results of the binary logistic regression analysis for NC and OCI indicate carrier status of *APOE* ε4 and total SCD-Q9 score were independent risk factors of OCI [OR: 2.050, 95% CI (confidential interval): 1.161–3.620, *p* = 0.013, and OR: 1.444, 95% CI: 1.285–1.622, *p* < 0.001, respectively]. When we split OCI group into MCI and AD dementia groups, only total SCD-Q9 score was the independent risk factor of MCI [OR: 1.390, 95% CI: 1.232–1.568, *p* < 0.001], whereas carrier status of *APOE* ε4 did not show any relationship (*p* = 0.191).

Our results also showed that carrying the *APOE* ε4 allele (OR: 1.960, 95% CI: 1.184–3.243, *p* = 0.009) was a risk factor for OCI compared with SCDs, whereas scores of SCD-Q9 did not show any relationship (*p* = 0.153). When we split OCI group into MCI and AD dementia groups, carrying the *APOE* e4 allele and total SCD-Q9 score were not affecting factors for MCI compared with SCD (*p* = 0.265 and *p* = 0.792, respectively).

### Receiver Operating Characteristics of Normal Control, SCDs, and Objective Cognitive Impairment Groups

**FIGURE 1:**
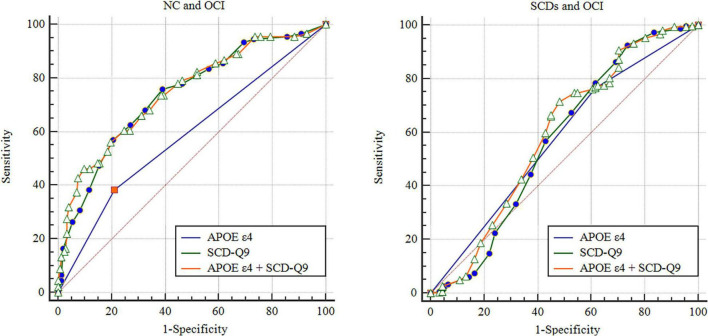
Receiver operating characteristic of NC, SCDs, and OCI groups. NC, normal control; SCDs, subjective cognitive decline_*with subtle cognitive decline*_; OCI, objective cognitive impairment; SCD-Q9, Subjective Cognitive Decline-Questionnaire 9; ROC, receiver operating characteristic.

Based on the results of logistic analysis, we calculated the AUCs of *APOE* ε4 itself for group discrimination, which was 0.587 (95% CI: 0.517–0.658, *p* = 0.014) between NC and OCI groups and 0.575 (95% CI: 0.506–0.644, *p* = 0.031) between SCDs and OCI groups (see details in Figure 1). In the female subgroup, the AUCs were 0.634 (95% CI: 0.537–0.731, *p* = 0.007) and 0.506 (95% CI: 0.445–0.567, *p* = 0.846) between NC and OCI groups and SCDs and OCI groups, respectively. In males, the AUCs were 0.550 (95% CI: 0.447–0.653, *p* = 0.337) between NC and OCI groups and 0.542 (95% CI: 0.455–0.630, *p* = 0.342) between SCDs and OCI groups.

The AUCs of SCD-Q9 alone were 0.727 (95% CI: 0.682–0.771, *p* < 0.001) for the NC and SCDs groups, 0.738 (95% CI: 0.678–0.799, *p* < 0.001) for the NC and OCI groups, and 0.571 (95% CI: 0.494–0.648, *p* = 0.040) for the SCDs and OCI groups, respectively (see details in Figure 1).

Then, we included scores of SCD-Q9 and *APOE* ε4 carrier status together in the model and calculated AUCs for group discrimination. The AUCs increased to 0.747 (95% CI: 0.685–0.808, *p* < 0.001) for the NC and OCI groups, and 0.593 (95% CI: 0.518–0.668, *p* < 0.001) for the SCDs and OCI groups, respectively (see details in Figure 1). In females, the AUCs increased to 0.758 (95% CI: 0.670–0.846, *p* < 0.001) for the NC and OCI groups and 0.707 (95% CI: 0.649–0.764, *p* < 0.001) for the SCDs and OCI groups. In males, the AUCs were 0.753 (95% CI: 0.668–0.839, *p* < 0.001) for the NC and OCI groups and 0.756 (95% CI: 0.684–0.827, *p* < 0.001) for the SCDs and OCI groups.

We also performed binary logistic regression analysis and calculated the AUCs of all the related factors [including demographics (age, gender, and education years), HAMD and HAMA scores] for NC, SCDs, and OCI groups (see details in Supplementary Material 2,3).

## Discussion

In the current study, we first reported the distribution characteristics of *APOE* ε4 alleles in a Chinese memory clinic with a larger cohort. To the best of our knowledge, this is also the first study to reveal the associations of *APOE* ε4 and SCD-Q9 scores in subjective and OCI diagnosed based on the combination of SCD-I (Jessen et al., 2014) and the Jak/Bondi standards (Edmonds et al., 2015). These results will help us better understand the variation in the unfavorable effect of *APOE* ε4 on the disease progression of AD and identify individuals with higher risk of cognitive decline in order to intervene earlier and treat more effectively.

Our findings showed that the frequency of *APOE* ε4 was 13.1%, following the *APOE* ε3 (78.5%), as the second most common allele. This is similar to the previous report based on the worldwide distribution (Farrer et al., 1997), showing *APOE* ε4 and *APOE* ε3 with a frequency of 13.7 and 77.9%, respectively. However, compared with a study in China by Li et al. (2002), we reported a higher frequency of *APOE* ε4 (13.1 vs. 9.7%), which may be due to the different recruitment protocol and more patients with OCI participating in our study. Participants with more memory impairment were included in our investigation, which may have resulted in a higher carrier rate of *APOE* ε4. Recently, a higher frequency of *APOE* ε4 alleles (19.6%) was reported in a population with cognitive impairment from a Chinese memory clinic, which supports our assumption (Wang et al., 2014). Moreover, compared with Oceania, South Africa, and Europe, such as Australia (26.0%) (Kamboh et al., 1991), Khoi San (37.0%) (Sandholzer et al., 1995), and NE England (15.4%), respectively (Mastana et al., 1998), a numeric lower frequency of *APOE* ε4 alleles was found in our study, which was also consistent with the previous conclusions that the frequency of *APOE* ε4 varied among different ethnicities and that Asian populations have a relatively lower ε4 frequency (Singh et al., 2006; Eisenberg et al., 2010). This also may be one of the reasons that the worldwide distribution of *APOE* ε4 appeared at a relatively low level.

Second, we analyzed the distribution characteristics of the *APOE* ε4 alleles in different demographics. The results showed that their frequencies did not vary with age, which was in agreement with the previous studies (Nakayama and Kuzuhara, 1999; Thelma et al., 2001). However, results from Alzheimer’s Disease Neuroimaging Initiative (ADNI) cohorts and Uniform Data Set of the Alzheimer’s Disease (UDS) Centers and Australian Imaging, Biomarkers and Lifestyle Flagship Study of Ageing (AIBL), both demonstrated significant correlations between *APOE* ε4 and aging (Heffernan et al., 2016). This contradiction may be attributed to uncertainties inherent in the study design. For instance, several confounding factors of AD in the UDS and ADNI datasets, such as severe heart disease and diabetes, were excluded from our study. Studies have also reported that the frequencies of *APOE* ε4 decreased with aging (Jian-Gang et al., 1998; Bonham et al., 2016; Liu and Caselli, 2018; Bellou et al., 2020; Le Couteur et al., 2020) and the risk mainly declined after 75 years of age (Bickeboller et al., 1997; Jian-Gang et al., 1998; Bonham et al., 2016; Liu and Caselli, 2018). In the current study, only 9.3% of individuals older than 75 years agreed to participate in our investigation which may have contributed to this discrepancy. At present, knowledge of education and *APOE* ε4 alleles is limited. To the best of our knowledge, only one study previously investigated this association. Their results indicated that the relationship was not correlated (Caselli et al., 2009) which is consistent with findings from the current study. Lastly, we did not find a significant difference between gender and *APOE* ε4 allele distribution, which is consistent with previous findings (Combarros et al., 1998; Vaisi-Raygani et al., 2007; Tsolaki et al., 2018).

Finally, our study reported the distribution of *APOE* ε4 alleles in different cognitive groups and their associations with the SCD-Q9 scores. The most robust findings have demonstrated that the presence of the *APOE* ε4 allele imparts a genetic risk for the development of cognitive impairment, specifically that related to AD and vascular dementia (Allan and Ebmeier, 2011; Pink et al., 2015; Chen et al., 2016a; Jiang et al., 2016; Liu et al., 2016). Ali et al. (2018) suggested that the frequencies of carrying the *APOE* ε4 allele were comparable between healthy controls and SCD samples but were significantly higher in objectively impaired samples (i.e., MCI and AD dementia). In our study, the frequencies of *APOE* ε4 allele in the NC and SCDs groups were lower than that of the OCI groups in the total population and the female subgroup, but we did not find a difference between the NC and SCDs groups, which was in line with Ali et al.’s (2018) conclusion. In addition, a previous study found that individuals with subjective memory complaints (SMC) with no objective memory impairment did not differ from the NC group in terms of the frequency of *APOE* ε4 alleles (Lautenschlager et al., 2005). The results of our study did not provide supportive evidence for a positive association between *APOE* ε4 and SCD. However, a study has previously reported higher *APOE* ε4 frequency in the SCD group than in normal controls (Jessen et al., 2018), and this inconsistency may be due to different study populations (i.e., gender and ethnicity) and diagnostic criteria. The German Center for Neurodegenerative Diseases (DZNE)-Longitudinal Cognitive Impairment and Dementia Study (DELCODE) enrolled subjects who speak fluent German and defined SCD based on the SCD-I diagnostic frame, which differs from ours. Also, the relatively higher prevalence of SCD but lower conversion rate to OCI in China due to low level of education and income (Wang et al., 2000; Rohr et al., 2020; Si et al., 2020) may result in different conclusions.

In our study, a significant difference in SCD-Q9 scores was found between *APOE* ε4 allele carriers and non-carriers in OCI in the total population and female subgroup. These results indicate that *APOE* ε4 alleles may be partially reflected in SCD-Q9 scores in patients with OCI in total population and females. Meanwhile, *APOE* ε4 allele carriers also presented a higher score of SCD-Q9 than non-carriers in the total population and SCDs group, but the differences were not significant. This is the first attempt to explore the relationship between *APOE* ε4 and SCDs diagnosed based on the combination standards of SCD-I and Jak/Bondi, which needs to be further verified by follow-up studies. Further attention should also be paid to other ethnicities and cohorts to verify this association in the future. Finally, the results of logistic regression analysis showed that *APOE* ε4 allele was risk factor for the OCI group (MCI and AD dementia) but not for the NC and SCDs groups, although the predictive powers were smaller. However, we found AUCs of SCD-Q9 alone were 0.727 for the NC and SCDs groups, 0.738 for the NC and OCI groups, and 0.571 for the SCDs and OCI groups, respectively. When *APOE* ε4 carrier status and SCD-Q9 scores together were added to the model, the AUC increased to 0.747 for NC and OCI groups, and 0.593 for SCDs and OCI groups, suggesting that the predictive power of *APOE* ε4 is limited, especially when OCI group was split into MCI and dementia groups, their discriminating powers for MCI and NC were marginal, but could be increased by combining with scores of SCD-Q9. Previous studies reported that *APOE* ε4 status appeared to be a more predictive risk factor for progression from MCI to AD dementia than family history, age, gender, or education (Fleisher et al., 2007), but it was only a useful predictor of progression from 70 to 85 years of age while controlling for education, memory scores, and gender (Devanand et al., 2005). Another study reported that ε4 carriers with SMC showed altered AD-related cerebrospinal fluid and fluorodeoxyglucose-positron emission tomography (PET) measures (Mosconi et al., 2008); In addition, they demonstrated that aging, *APOE* ε4, and SMC were associated with high Aβ burden, indicating that selection based on the presence of SMC and *APOE* ε4 may help identify healthy elderly participants with high Aβ burden eligible for secondary prevention trials (Zwan et al., 2016). As encouraging as these results may be, the exact role played by *APOE* ε4 in the development of AD dementia or other OCI continues to be unclear due to a lack of convergent evidence and considerable sample heterogeneity (Ali et al., 2018). Consequently, further investigation is warranted before *APOE* ε4 genetic testing can be recommended for wide-scale clinical adoption as a viable diagnostic tool for pathological cognitive decline.

It should be noted that there were obvious limitations associated with this study. (1) Our study is a cross-sectional survey, and follow-up studies should be performed to further confirm the conclusions; (2) the diagnosis of subjective and OCI was not validated by other tests. For instance, it lacks the completeness of Aβ-PET, cerebrospinal fluid tau, or Aβ examinations, given that only parts of the included population underwent Aβ-PET; (3) finally, this study focused on *APOE* ε4 alleles; thus, no evidence was provided for other related biomarkers and imaging approaches; and (4) the small sample size of OCI group in current study restricts us to further confirm the relationship between *APOE* ε4 and SCD-Q9 after control the amount of cognitive impairment, and a larger cohort with MCI and mild AD dementia patients was needed to verify our conclusion.

## Conclusion

In summary, we reported the distribution characteristics of *APOE* ε4 alleles in different demographics and levels of cognition, and their associations with scores of SCD-Q9 with a larger cohort from a Chinese memory clinic. The findings of this study indicate that clinicians should be attentive to the distributed variation of *APOE* ε4 alleles and their unfavorable effects on OCI with SCD complaints, but their additional contribution to SCD-Q9 scores is marginal in discriminating individuals with cognitive impairment from normal controls.

## Data Availability Statement

The original contributions presented in the study are included in the article/Supplementary Material, further inquiries can be directed to the corresponding authors.

## Ethics Statement

The studies involving human participants were reviewed and approved by the Xuanwu Hospital Capital Medical University. The patients/participants provided their written informed consent to participate in this study.

## Author Contributions

LH: conceiving, implementing, statistics, and writing the manuscript. JJ: revising the manuscript. YX and YH: conceiving and revising the manuscript. YH: providing funding support. All authors contributed to the article and approved the submitted version.

## Conflict of Interest

The authors declare that the research was conducted in the absence of any commercial or financial relationships that could be construed as a potential conflict of interest.

## Publisher’s Note

All claims expressed in this article are solely those of the authors and do not necessarily represent those of their affiliated organizations, or those of the publisher, the editors and the reviewers. Any product that may be evaluated in this article, or claim that may be made by its manufacturer, is not guaranteed or endorsed by the publisher.
