# Supplementary material for: APOE ε4 Allele Distribution and Association With Scores of Subjective Cognitive Decline Questionnaire 9 in a Large Chinese Memory Clinic Cohort
Source: Front Neurosci. 2022 Jun 3;16:829031. doi: 10.3389/fnins.2022.829031 (PMC9204235; doi:10.3389/fnins.2022.829031)
Supplement: Supplementary file 1 [file Data_Sheet_1.pdf]

Supplementary Material

1 Supplementary Data

1. Demographic characteristics and scores of SCD-Q9 in NC, SCD$_{with\ subtle\ cognitive\ decline}$ (SCDs) and OCI groups

There was a significant difference in gender between NC, SCDs and OCI groups ($p<0.05$). Further pairwise comparisons showed that the percentage of males in the SCDs group was lower than that in the NC ($p<0.001$) and OCI groups ($p<0.001$). However, we did not find a significant difference between the NC and OCI groups ($p=0.107$).

The difference in age among the three groups was also significant ($p<0.05$). For pairwise comparison, we found those in the OCI group with the age of 67.8 ± 8.07 years, were older than NC (65.2 ± 6.23, $p=0.002$) and SCDs (64.9 ± 6.62, $p<0.001$) groups, but no significant difference was found between NC and SCDs groups ($p=0.673$).

In addition, the education levels in the NC, SCDs, and OCI groups were significantly different ($p<0.05$). The results showed that the education years of the NC (12.4±3.18) and SCDs (12.8±3.07) groups were higher than that in the OCI (11.4±3.50) group by pairwise comparisons (NC vs OCI, $p=0.010$; SCDs vs OCI, $p<0.001$). However, we did not find a significant difference between the NC and SCDs groups ($p=0.124$) .

For SCD-Q9 scores, the three groups showed significant differences ($p<0.05$). The OCI group demonstrated higher SCD-Q9 scores (5.31 ± 2.34) compared to the SCDs group (4.94 ± 1.64, $p<0.001$) and NC group (3.24 ± 2.26, $p<0.001$). However, no significant difference was found between SCDs and OCI ($p=0.120$).

Finally, we compared the HAMD and HAMA scores between the three groups, and the results showed that the differences were also significant ($p<0.001$). Compared with NC group (2.93 ± 3.68), the OCI (4.91 ± 5.57, $p<0.001$) and SCDs (4.79 ± 4.60, $p<0.001$) groups had higher HAMD scores. For HAMA scores, the NC group (3.59 ± 3.85) also scored lower than the SCDs (5.58 ± 5.00, $p<0.001$) and OCI (4.76 ± 5.01, $p=0.039$) groups. No significant differences of HAMD and HAMA were found between SCDs and OCI groups ($p=0.816$ and $p=0.136$, respectively).(see table S1)

Table S1. The demographic characteristics, scores of SCD-Q9, HAMD and HAMA in NC, SCDs and OCI groups

Variables	NC (N=243)	SCDs (N=298)	OCI (N=91, MCI=77, AD dementia=14)	*p*
Males n(%)	99(40.7)	73(24.5)	46(50.5)	<0.001
Age (x±s)	65.2±6.23	64.9±6.62	67.8±8.07	0.002
Education (x±s)	12.4±3.18	12.8±3.07	11.4±3.50	0.001
Scores of SCD-Q9 (x±s)	3.24±2.26	4.94±1.64	5.31±2.34	<0.001
HAMD (x±s)	2.93±3.68	4.79±4.60	4.91±5.57	<0.001
HAMA (x±s)	3.59±3.85	5.58±5.00	4.76±5.01	<0.001

NC: Normal control; **SCDs:** Subjective cognitive decline with subtle cognitive decline; **OCI:** Objective Cognitive Impairment; **SCD-Q9:** Subjective cognitive decline-questionnaire9; **MCI:** Mild cognitive impairment; **AD:** Alzheimer disease; **HAMD:** Hamilton depression scale; **HAMA:** Hamilton anxiety scale.

2. Logistic regression models for NC, SCDs and OCI groups

The results of the binary logistic regression analysis for NC and SCDs showed that the female gender (OR: 1.860, 95% CI: 1.230−2.811), higher education (OR: 1.085, 95% CI: 1.020−1.155), and SCD-Q9 scores (OR: 1.497, 95% CI:1.346−1.666) were important risk factors for SCDs (p<0.05); however, we did not find significant effects for age, *APOE* ε4-positive status, and HAMD and HAMA scores (p>0.05) (see Table S2).

For NC and OCI, gender, years of education, SCD-Q9 scores, being an *APOE* ε4 carrier, and HAMD scores were significant factors. SCD-Q9 scores, HAMD scores, and being an *APOE* ε4 carrier were important risk factors for OCI (OR: 1.417, 95% CI: 1.247− 1.609; OR: 1.136, 95% CI: 1.039−1.241; and OR: 2.302, 95% CI: 1.256−4.221; respectively; p<0.05), whereas the female gender (OR: 0.439, 95% CI: 0.246−0.786) and higher education years (OR: 0.891, 95% CI:

0.818−0.972) showed protective effects ($p<0.05$). Moreover, we did not find a significant effect for age and HAMA scores on OCI ($p>0.05$) (see Table S3).

Our results also showed that the female gender (OR: 0.274, 95% CI: 0.159−0.472) and higher education (OR: 0.812, 95% CI: 0.741−0.890) were protective factors for OCI compared to SCDs ($p<0.05$), whereas aging (OR: 1.052, 95% CI: 1.010−1.096) and being an *APOE* ε4 carrier (OR: 2.232, 95% CI: 1.287−3.872) showed risk effects ($p<0.05$). SCD-Q9, HAMD, and HAMA scores did not show significant effects on OCI ($p>0.05$) (see Table S4).

Table S2. Logistic regression model for NC and SCDs groups

Variables	B	S.E.	Wald	OR	95% C.I	*p*
Gender	0.620	0.211	8.658	1.860	1.230, 2.811	0.003
Age	-0.002	0.015	0.016	0.998	0.968, 1.028	0.899
Education	0.082	0.032	6.645	1.085	1.020, 1.155	0.010
Scores of SCD-Q9	0.404	0.054	55.015	1.497	1.346, 1.666	0.000
Carriers of ApoE ε4	0.151	0.231	0.425	1.162	0.739, 1.828	0.514
HAMD	0.045	0.035	1.710	1.046	0.978, 1.120	0.191
HAMA	0.005	0.033	0.024	1.005	0.942, 1.072	0.876

NC: Normal Control; **SCDs:** Subjective cognitive decline with subtle cognitive decline; **SCD-Q9:** Subjective cognitive decline-questionnaire 9. **OR:** Odds ratios; **CI:** Confidential Interval; **HAMD:** Hamilton depression scale; **HAMA:** Hamilton anxiety scale.

Table S3. Logistic regression model for NC and OCI groups

Variables	B	S.E.	Wald	OR	95% CI	*p*
Gender	-0.823	0.297	7.677	0.439	0.246, 0.786	0.006
Age	0.038	0.022	3.061	1.038	0.995, 1.083	0.080
Education	-0.115	0.044	6.847	0.891	0.818, 0.972	0.009
Scores of SCD-Q9	0.348	0.065	28.773	1.417	1.247, 1.609	0.000
Carriers of ApoE ε4	0.834	0.309	7.274	2.302	1.256, 4.221	0.007
HAMD	0.127	0.045	7.815	1.136	1.039, 1.241	0.005
HAMA	-0.088	0.050	3.121	0.916	0.831, 1.010	0.077

NC: Normal Control; **OCI:** Objective Cognitive Impairment; **SCD-Q9:** Subjective cognitive decline-questionnaire 9; **OR:** Odds ratios; **CI:** Confidential Interval; **HAMD:** Hamilton depression scale; **HAMA:** Hamilton anxiety scale.

Table S4. Logistic regression model for SCDs and OCI groups

Variables	B	S.E.	Wald	OR	95% CI	*p*
Gender	-1.295	0.278	21.662	0.274	0.159, 0.472	0.000
Age	0.050	0.021	5.846	1.052	1.010, 1.096	0.016
Education	-0.208	0.047	20.048	0.812	0.741, 0.890	0.000
Scores of SCD-Q9	0.113	0.076	2.203	1.119	0.965, 1.299	0.138
Carriers of ApoE ε4	0.803	0.281	8.172	2.232	1.287, 3.872	0.004
HAMD	0.058	0.039	2.165	1.059	0.981, 1.144	0.141
HAMA	-0.079	0.041	3.595	0.924	0.852, 1.003	0.058

SCDs: Subjective cognitive decline with subtle cognitive decline; **OCI:** Objective Cognitive Impairment; **SCD-Q9:** Subjective cognitive decline-questionnaire 9; **OR:** Odds ratios; **CI:** Confidential Interval; **HAMD:** Hamilton depression scale; **HAMA:** Hamilton anxiety scale.

3.ROCs of NC, SCDs and OCI groups

Based on the outcome of logistic analysis, we analyzed all these related factors (including age, gender, education years, scores of SCD-Q9 and *APOE* ε4 status) together in the model and calculated AUCs for group discrimination. The AUCs greatly increased to 0.752 (95% CI: 0.710−0.794, p<0.001) for the NC and SCDs groups, 0.798 (95% CI: 0.744−0.852, p<0.001) for NC and OCI groups, and 0.762 (95% CI: 0.707−0.818, p<0.001) for SCDs and OCI groups, respectively.

4.Neuropsychological characteristics and SCD-Q9 scores of NC, SCDs and OCI groups

The results showed that differences in all neuropsychological scores among the three groups (NC, SCDs and OCI) were significant (p<0.001). For pairwise comparison, we found OCI group scored lower of Mini-Mental State Examination (MMSE), Montreal Cognitive Assessment (MoCA), Auditory Verbal Learning Test- Long Delay Recall (AVLT-LR), AVLT- Recognition, Animal Fluency Test (AFT) and Boston Naming Test (BNT) than that in the NC and SCDs groups, but scored higher on Shape Trail Making Test-A (STT-A) and Shape Trail Making Test-B (STT-B) than the other two groups (p<0.001). Although SCDs groups scored lower in several neuropsychological scores (MMSE, AVLT-LR, AVLT-Recognition and BNT) than that of NC group, we did not find any significant difference between them (p＞0.05). There were also no significant difference in

MoCA, STT-A, STT-B and AFT between NC and SCDs groups ($p > 0.05$). For Hamilton Anxiety Scale (HAMA) and Hamilton Depression Scale (HAMD), the NC group scored higher than SCDs and OCI groups ($p<0.05$), and no significant difference was found between SCDs and OCI groups ($p>0.05$).

We also compared the scores of SCD-Q9 between groups, the difference between NC, SCDs and OCI groups was significant ($p<0.001$). For pairwise comparison, SCDs and OCI scored higher on SCD-Q9 than the NC group ($p<0.001$). (please see details in supplementary Table S5).

Table S5. Neuropsychological characteristics and SCD-Q9 scores of NC, SCDs and OCI groups

Variables	Groups			*p*
	NC	SCDs	OCI	
MMSE	28.8±1.31	28.7±5.03	25.6±3.77	＜0.001
MoCA	25.9±2.51	25.9±2.48	20.5±4.35	＜0.001
AVLT-LR	7.6±2.02	7.4±2.25	3.4±2.95	＜0.001
AVLT-Recognition	22.6±1.46	22.5±1.64	18.6±3.41	＜0.001
STT-A	59.3±20.18	56.7±17.92	98.9±44.40	＜0.001
STT-B	139.2±42.65	136.6±37.88	227.4±101.15	＜0.001
AFT	19.5±4.16	19.5±4.59	14.6±4.15	＜0.001
BNT	25.5±2.84	25.2±2.77	21.4±4.61	＜0.001
HAMA	3.6±3.85	5.6±5.00	4.8±5.01	＜0.001
HAMD	2.9±3.68	4.8±4.60	4.9±5.57	＜0.001

SCD-Q9	3.2±2.26	4.9±1.64	5.3±2.34	＜0.001

NC: Normal control; **SCDs:** Subjective cognitive decline with subtle cognitive decline; **OCI:** Objective Cognitive Impairment; **SCD-Q9:** Subjective cognitive decline-questionnaire9; **MMSE:** Mini-Mental State Examination; **MoCA:** Montreal Cognitive Assessment; **AVLT-LR:** Auditory Verbal Learning Test Long Delay Recall; **STT-A:** Shape Trail Making Test-A; **STT-B:** Shape Trail Making Test-B; **AFT:** Animal Fluency Test; **BNT:** Boston Naming Test; **HAMA:** Hamilton Anxiety Scale; **HAMD:** Hamilton Depression Scale.

5. ***Neuropsychological characteristics of carriers and non-carriers of ApoE ε4***

For carriers and non-carriers of ε4 groups, we found carriers scored lower of MMSE, MoCA, AVLT-LR, AVLT- Recognition and AFT than non-carriers (p<0.001). However, we did not find any significant difference in STT-A, STT-B, BNT, HAMA and HAMD between them (p>0.05).

Table S6. Neuropsychological characteristics of carriers and non-carriers of ApoE ε4

Variables	Groups		*p*
	non-carriers ε4	carriers ε4	
MMSE	28.6±3.97	27.0±3.65	＜0.001
MoCA	25.4±2.95	24.0±4.38	＜0.001
AVLT-LR	7.2±2.45	6.1±3.20	＜0.001
AVLT-Recognition	22.2±1.92	21.2±3.29	＜0.001
STT-A	62.9±27.17	66.4±31.01	0.192
STT-B	147.9±62.82	157.2±55.75	0.107
AFT	19.2±4.63	17.6±4.67	＜0.001
BNT	24.8±3.36	24.6±3.57	0.548

HAMA	4.6±4.54	5.2±5.07	0.165
HAMD	4.0±4.38	4.4±4.92	0.404

MMSE: Mini-Mental State Examination; **MoCA:** Montreal Cognitive Assessment; **AVLT-LR**: Auditory Verbal Learning Test Long Delay Recall; **STT-A:** Shape Trail Making Test-A; **STT-B:** Shape Trail Making Test-B; **AFT:** Animal Fluency Test; **BNT:** Boston Naming Test; **HAMA:** Hamilton Anxiety Scale; **HAMD:** Hamilton Depression Scale.

6. *Neuropsychological characteristics of carriers and non-carriers of ApoE ε4 in NC group*

For carriers and non-carriers of ε4 groups, we did not find any significant difference in all neuropsychological scores in NC group (*p*>0.05). (please see details in S7)

Table S7. Neuropsychological characteristics of carriers and non-carriers of ApoE ε4 in NC group

Variables	Groups		*p*
	non-carriers ε4	carriers ε4	
MMSE	28.8±1.37	29.0±1.05	0.138
MoCA	26.0±2.52	25.4±2.45	0.807
AVLT-LR	7.7±1.98	7.5±2.19	0.609
AVLT-Recognition	22.6±1.44	22.4±1.53	0.343
STT-A	58.8±19.00	61.1±24.30	0.199
STT-B	137.3±43.96	146.5±36.84	0.165
AFT	19.7±4.13	18.6±4.16	0.965

BNT	25.5±2.82	25.4±2.95	0.392
HAMA	3.4±3.90	4.2±3.67	0.763
HAMD	2.8±3.44	3.5±4.47	0.056

NC: Normal control; **MMSE:** Mini-Mental State Examination; **MoCA:** Montreal Cognitive Assessment; **AVLT-LR**: Auditory Verbal Learning Test Long Delay Recall; **STT-A:** Shape Trail Making Test-A; **STT-B:** Shape Trail Making Test-B; **AFT:** Animal Fluency Test; **BNT:** Boston Naming Test; **HAMA:** Hamilton Anxiety Scale; **HAMD:** Hamilton Depression Scale.

7. ***Neuropsychological characteristics of carriers and non-carriers of ApoE ε4 in SCDs group***

For carriers and non-carriers of ε4 groups, we did not find any significant difference in all neuropsychological scores in SCDs group (p>0.05). (please see details in S8)

Table S8. Neuropsychological characteristics of carriers and non-carriers of ApoE ε4 in SCDs group

Variables	Groups		*p*
	non-carriers ε4	carriers ε4	
MMSE	28.9±5.62	28.0±2.23	0.840
MoCA	25.9±2.42	25.7±2.69	0.462
AVLT-LR	7.5±2.27	7.2±2.20	0..531
AVLT-Recognition	22.5±1.55	22.5±1.90	0.288
STT-A	56.7±18.32	56.9±16.77	0.315
STT-B	135.7±36.65	139.5±41.58	0.064

AFT	19.6±4.68	19.0±4.28	0.295
BNT	25.0±2.88	25.6±2.38	0.164
HAMA	5.6±5.00	5.5±5.05	0.903
HAMD	4.9±4.70	4.6±4.31	0.646

SCDs: Subjective cognitive decline with subtle cognitive decline; **MMSE:** Mini-Mental State Examination; **MoCA:** Montreal Cognitive Assessment; **AVLT-LR**: Auditory Verbal Learning Test Long Delay Recall; **STT-A:** Shape Trail Making Test-A; **STT-B:** Shape Trail Making Test-B; **AFT:** Animal Fluency Test; **BNT:** Boston Naming Test; **HAMA:** Hamilton Anxiety Scale; **HAMD:** Hamilton Depression Scale.

8. *Neuropsychological characteristics of carriers and non-carriers of ApoE ε4 in OCI group*

For carriers and non-carriers of ε4 groups, we found carriers scored higher of MoCA, HAMA and HAMD than non-carriers ($p<0.05$). However, we did not find any significant difference in AVLT-LR, AVLT-Recognition, STT-A, STT-B, AFT and BNT between them ($p>0.05$).

Table S9. Neuropsychological characteristics of carriers and non-carriers of ApoE ε4 in OCI group

Variables	Groups		*p*
	non-carriers ε4	carriers ε4	
MMSE	25.2±5.21	25.9±3.20	0.470
MoCA	20.3±4.62	21.4±2.89	0.042
AVLT-LR	3.8±3.10	2.6±2.27	0.101
AVLT-Recognition	18.9±3.65	18.1±2.40	0.145

STT-A	100.2±40.32	85.2±41.36	0.756
STT-B	223.8±88.42	207.2±74.61	0.820
AFT	14.6±4.20	15.4±3.59	0.366
BNT	21.3±4.17	22.4±4.88	0.389
HAMA	4.2±3.55	6.5±7.81	0.003
HAMD	4.5±4.82	6.0±7.43	0..033

OCI: Objective Cognitive Impairment; **MMSE:** Mini-Mental State Examination; **MoCA:** Montreal Cognitive Assessment; **AVLT-LR**: Auditory Verbal Learning Test Long Delay Recall; **STT-A:** Shape Trail Making Test-A; **STT-B:** Shape Trail Making Test-B; **AFT:** Animal Fluency Test; **BNT:** Boston Naming Test; **HAMA:** Hamilton Anxiety Scale; **HAMD:** Hamilton Depression Scale.
